# Determinants for Neoantigen Identification

**DOI:** 10.3389/fimmu.2019.01392

**Published:** 2019-06-24

**Authors:** Andrea Garcia-Garijo, Carlos Alberto Fajardo, Alena Gros

**Affiliations:** Tumor Immunology and Immunotherapy, Vall d'Hebron Institute of Oncology (VHIO), Barcelona, Spain

**Keywords:** cancer, immunotherapy, neoantigen, vaccine, T-cell therapy, review

## Abstract

All tumors accumulate genetic alterations, some of which can give rise to mutated, non-self peptides presented by human leukocyte antigen (HLA) molecules and elicit T-cell responses. These immunogenic mutated peptides, or neoantigens, are foreign in nature and display exquisite tumor specificity. The correlative evidence suggesting they play an important role in the effectiveness of various cancer immunotherapies has triggered the development of vaccines and adoptive T-cell therapies targeting them. However, the systematic identification of personalized neoantigens in cancer patients, a critical requisite for the success of these therapies, remains challenging. A growing amount of evidence supports that only a small fraction of all tumor somatic non-synonymous mutations (NSM) identified represent *bona fide* neoantigens; mutated peptides that are processed, presented on the cell surface HLA molecules of cancer cells and are capable of triggering immune responses in patients. Here, we provide an overview of the existing strategies to identify candidate neoantigens and to evaluate their immunogenicity, two factors that impact on neoantigen identification. We will focus on their strengths and limitations to allow readers to rationally select and apply the most suitable method for their specific laboratory setting.

## Introduction

Cancer arises as a result of the accumulation of DNA damage and genetic alterations. Mutated gene products can be processed and presented in the form of small peptides on major histocompatibility complex (MHC) molecules of tumor cells and some can elicit T-cell responses. Such immunogenic mutated peptides, referred to as neoantigens, are emerging as promising targets to develop personalized clinical interventions.

Awareness that T cells can target cancer neoantigens is not novel. The dissection of the molecular nature of neoantigens derived from tumor variants induced through exposure to chemical carcinogens was first performed in mice in the late 1980s. The coding regions of three tumor-rejection antigens identified all contained mutations that changed one amino acid in proteins that were ubiquitously expressed (1–3). Importantly, the corresponding wild-type peptides were not immunogenic. The first strategy employed to identify human T-cell reactivities to neoantigens involved the laborious screening of cytotoxic tumor-reactive lymphocytes for recognition of tumor cDNA library pools by transfecting them along with the proper human leukocyte antigen (HLA) restriction element into transfectable target cells (4). In addition, neoantigen-specific responses dominated compared to responses targeting shared antigens in a patient with melanoma suggesting a greater contribution of neoantigen-specific T cells to antitumor immunity (5). The immunotherapeutic potential of targeting neoantigens was already acknowledged at the time. Neoantigens are specifically expressed by tumor cells and immunotherapeutic targeting of these antigens should be safe. In addition, neoantigens elicit T-cell responses that are not subject to central tolerance in the thymus, suggesting that immune responses against these antigens should be more potent. However, the difficulties of identifying such personalized peptides and T cells were daunting.

Recent technological innovations have enabled the systematic dissection of the personalized T-cell response targeting the tumor mutanome. Retrospective studies have shown that patients that exhibited complete tumor regressions following tumor-infiltrating lymphocyte (TIL) therapy have a higher tumor mutation burden (6) and TILs from responders frequently contain neoantigen-specific lymphocytes (7–11). Antibodies targeting the CTLA-4 and PD-1 pathways have shown the greatest clinical activity in tumor histologies with higher mutation load and brisk T-cell infiltrates such as metastatic melanoma, non-small-cell lung carcinoma (NSCLC), bladder cancer, and tumors with DNA-mismatch-repair deficiencies (12). Even within one same tumor histology, patients whose tumors have a higher mutation load display greater clinical benefit following treatment with immune checkpoint inhibitors (13–15), and this association has been observed across multiple cancer types (16). It is worth noting that a few retrospective studies have also reported a lack of correlation between high tumor mutational burden and clinical benefit in some tumor types (17, 18). Overall, the majority of clinical data are consistent with the hypothesis that higher mutation load is associated with higher likelihood to present neoantigens which can facilitate immune recognition of tumors as foreign.

The clinical correlative data coupled with the technological innovations to sequence tumors and to functionally dissect the personalized T-cell responses in cancer patients have spurred the development of immunotherapies targeting neoantigens. Active immunization strategies employed to treat patients rely on the identification of the non-synonymous mutations (NSM) by tumor whole exome sequencing (WES), *in silico* peptide HLA binding affinity prediction and prioritization of 10–20 candidate neoantigens, to manufacture RNA, synthetic long peptide or dendritic cell-based vaccines of unique composition. In one clinical trial, the vaccines also included candidate neoepitopes identified through elution from tumor cell-surface HLA-I molecules. Results reported thus far in patients with melanoma (19–21), and glioblastoma (22, 23) demonstrate that immunization with vaccines targeting neoantigens is feasible, safe and well tolerated. The melanoma trials reported clinical activity in some patients with detectable tumors at the time of vaccination, and some patients who progressed after vaccination and received anti-PD-1 therapy showed complete responses. More recently, two clinical studies of personalized neoantigen vaccines in patients with resected glioblastoma reported that, although vaccines triggered strong systemic T-cell responses, the majority of patients showed tumor recurrence. These first five clinical trials provide proof of principle that these approaches can enhance the frequency of pre-existing or *de novo* neoantigen-responses following immunization. However, induction of T cell responses were previously observed following immunization against shared antigens and this rarely translated into clinical benefit (24). Hence, significant challenges remain to be overcome including improvement of neoantigen selection, identifying the best route and method for immunization and overcoming intrinsic factors in the tumor microenvironment. However, the complete responses observed in post-vaccination melanoma patients receiving immune checkpoint inhibitors open a window of opportunity for the design of combinatorial approaches in the future.

In another approach different to vaccination, the infusion of large numbers of TILs targeting personalized cancer neoantigens have shown antitumor responses in selected cases of patients with cholangiocarcinoma (25), colorectal cancer (26), and breast cancer (27). This together with the prospective analyses of neoantigen reactivity in peripheral blood of melanoma, gastrointestinal (GI) and ovarian cancer patients suggesting that neoantigen-specific lymphocytes can be detected in the vast majority of patients screened (28–31), provide rationale to develop personalize T-cell based therapies targeting neoantigens.

## Determinants for Neoantigen Identification

Despite the increasing interest in clinical interventions targeting neoantigens, substantial challenges remain to enable a more precise identification of neoantigens that are relevant for patient treatment. RNA and synthetic peptide-based vaccines targeting neoantigens used to treat patients thus far lack prospective immunological testing of candidate neoantigens. Rather, these are selected largely based on *in silico* HLA-I binding affinity, making the selection of candidate neoantigens crucial for this therapeutic approach. Surprisingly, neoantigen vaccines reported appear to favor CD4^+^ over CD8^+^ responses. Moreover, only few of the patients immunized generated T-cell responses targeting the autologous melanoma cell lines (21), manifesting the limitations of *in silico* peptide HLA binding prediction alone to effectively identify neoantigens naturally processed and presented by the tumor.

Evidence arising from available studies is that only a small fraction of all NSM identified by tumor WES are actually processed, presented and recognized by T cells (8, 28, 29, 31–33). Many of these screenings interrogated the immunogenicity of all the candidate NSM identified by tumor WES, without using *in silico* prediction algorithms. Instead, they used a high through-put immunological screening method relying on the expression of all the mutated minigenes in the patient's own antigen presenting cells (APCs), which enables unbiased processing and presentation on the patient's own HLA-I and HLA-II molecules (described in more detail in section Unbiased Screening of All Candidate Neoantigens Identified by Tumor WES). Hence, the paucity of reactivities detected cannot be attributed to the limitations of *in silico* peptide prediction algorithms. Furthermore, the vast majority of selected candidate neoantigens identified in a tumor are also not effective in tumor rejection in mouse models (34, 35). Part of the reason that could explain this lack of immunogenicity lies in the fact that for a neoepitope to be recognized in a cancer patient, the T-cell receptor (TCR) repertoire of the patient needs to contain a TCR that specifically targets this peptide bound to a specific HLA allele. Although the TCR repertoire diversity in any given individual is thought to be capable of recognizing virtually any pathogen, this may not hold true for neoantigens which frequently differ from their wild-type counterparts only by one residue. Tumor heterogeneity is yet another potential factor that could hinder neoantigen identification.

**Figure 1:**
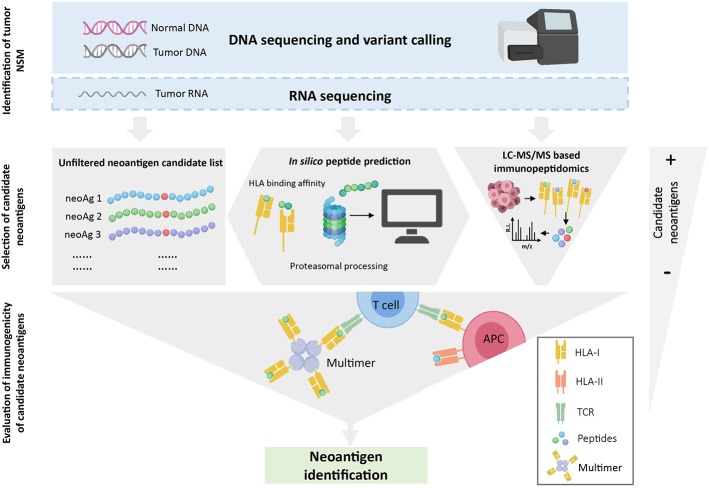
Overview of neoantigen identification using tumor WES. WES is performed on tumor and normal DNA to identify tumor-specific NSM. When available, RNAseq is used to select mutations that are expressed. Once NSM are identified, three strategies can be used to select the list of candidate neoantigens that will be assessed for immunogenicity. The gray-filled shapes depict how each selection strategy will dictate the final number of candidate peptides to be evaluated. Note that *in silico* prediction initially increases the number of potential candidates but, after a ranking-based selection of peptides, this number decreases substantially. Finally, the immunogenicity of the selected candidate peptides is evaluated with different immunological screening assays.

Estimating the exact number of neoantigen-specificities in a cancer patient is further complicated by the fact that *the absence of evidence is not evidence of absence*. Neoantigen identification is technically challenging and all the steps involved can impact on the outcome. Briefly, as depicted in Figure 1, WES from tumor and matched normal DNA is typically used to identify all cancer-specific NSM, all candidate neoantigens. The resulting neoepitope candidates can be further selected based on their likelihood to be processed and presented on the cell surface HLA molecules using *in silico* prediction algorithms or through selection of mutated epitopes bound to tumor cell-surface HLA molecules through immunopeptidomics. Finally, a variety of novel high-throughput immunological screening methods, with enhanced capacity to interrogate large numbers of candidate neoepitopes, are used to screen cancer-derived CD8^+^ and CD4^+^ T-cell populations of interest for neoantigen recognition. Given the technical complexity, it is entirely possible that a fraction of neoantigen-reactive lymphocytes are not detected due to limitations arising from the specific computational analysis performed to identify NSM from WES data, from the *in silico* peptide prediction algorithms, from the specific immunological screening assay and read-outs chosen and/or the limited frequency of neoantigen-specific TCR clonotypes within the chosen source of effector T-cell population used for the screening.

Overall, two critical factors can greatly influence the identification of *bona fide* immunogenic neoantigens: (1) the identification of candidate neoantigens, and (2) the evaluation of their immunogenicity. Given the emerging potential of neoantigens as therapeutic targets, and the crucial importance of these factors for neoantigen identification, the technical implications of these steps and advantages and disadvantages will be reviewed in detail.

## Identification of Candidate Neoantigens

The first element that can influence the identification of immunogenic neoantigens is the tumor-derived DNA and RNA sequencing and the computational analysis necessary to identify tumor-specific NSM.

### Identification of Tumor-Specific Non-synonymous Mutations

The process for discovering immunogenic neoantigens starts with the identification of all tumor somatic NSM. To date, this is generally done by mapping genetic alterations in the tumor genome using next generation sequencing (NGS). For each patient, Whole genome sequencing (WGS) or WES data from matched tumor and normal DNA is required. Following the alignment of normal and tumor reads to the human reference genome, somatic variants, which include single nucleotide variants (SNV), gene fusions and insertion or deletion variants (indels), can be detected using variant-calling algorithms. Multiple variant callers have been developed to date and each of them differ in their accuracy and sensitivity to detect different somatic variant types (i.e., SNV, gene fusions, or indels) (36). Indeed, several studies have compared distinct variant calling pipelines and reported substantial discrepancies in the detected variants from the same set of raw sequencing data (37, 38). Consequently, computational analysis pipelines commonly use more than one variant caller and select those somatic variants that are identified by several independent variant callers to reduce the number of false positives (39, 40). Integration of these pipelines will however not solve false-negative calls, which are somatic variants that, despite being potential neoantigens, will remain undetected, pointing out the need for improvement of sensitivity of variant calling algorithms. Of note, the performance of variant calling algorithms is directly related to the process of sequencing. Thus, current technical limitations of sequencing technology such as errors introduced by PCR amplification during library construction or mismapped reads can affect the accurate identification of somatic variants leading to detection of false variants (41). Tumor heterogeneity is an additional limitation for calling somatic variants with confidence, since it biases the detection of clonal over subclonal mutations due to differences in variant allele frequency, thus resulting in underrepresentation of somatic variants (41).

Although WES is currently the standard strategy used to identify candidate neoantigens, RNA sequencing (RNAseq) could alternatively be performed. RNAseq is currently used in combination with WES, to filter out those candidate neoantigens that do not exceed a selected threshold of gene expression. However, its usage should not be restricted to gene expression assessment as it provides additional information that might be essential for the identification of certain somatic variants that otherwise would remain undetected. For instance, low frequency somatic variants that might not be identified by WES could conversely be detected using RNAseq data if their read count is within the detection range (36). Moreover, as RNAseq surveys the entire transcriptome, it is the only method that allows the identification of peptides arising from RNA editing processes such as alternative splicing, gene fusions and post-transcriptional modifications (42, 43). Of note, unlike mutations identified using WES data, which can be assigned to the tumor but not normal DNA, alterations identified exclusively using tumor RNAseq data are not necessarily restricted to the tumor. Epitopes derived from edited RNA cannot immediately be considered candidate neoantigens until their expression in normal tissue has been ruled out. Nevertheless, the use of tumor RNAseq could provide a broader landscape of candidate neoantigens.

### Selection of Neoantigen Candidate Set of Interest

Following the identification of NSM, the neoantigen candidate set of interest can be (1) filtered using *in silico* peptide prediction algorithms, (2) selected based on the identification of specific neoepitopes eluted from tumor HLA through immunopeptidomics, or (3) left unfiltered to perform unbiased testing of all the neoantigens identified (Table 1).

**Table 1:** Strategies used for selection of candidate neoantigens.

**Strategy**	**Advantages**	**Disadvantages**
*In silico* peptide prediction and prioritization	Narrows down the number of candidate neoantigens Identifies minimal epitopes	Depends on accuracy of prediction algorithms Not optimal for HLA-II-presented peptides Less accurate predictions for low frequent HLA clonotypes
LC-MS/MS based immunopeptidomics	Direct identification of naturally presented HLA binding peptides Narrows down the number of candidate neoantigens Allows the identification of post-translational modified peptides and non-canonical neoantigens Identifies minimal epitopes	Limited sensitivity of mass spectrometry Biased toward detecting the more abundant peptides Relies on efficient peptide ionization and fragmentation Depends on HLA expression of tumor cells High amount of tumor tissue needed
List of all candidate neoantigens based on whole-exome sequencing data	Identification of all candidate neoantigens	Minimal epitope is not defined Limited feasibility in tumors with high mutation burden

*HLA, human leukocyte antigen*.

#### Selection of Candidate Neoantigens Using *in silico* Peptide Prediction

The advances in computational biology and immunology have led to the development of algorithms that allow to prioritize candidate peptides that are more likely to be presented on HLA-I based on biochemical and biophysical properties of most of the steps involved in peptide processing, transport and binding to HLA-I.

While peptide processing and transport prediction tools can give important information about the nature of peptides that are presented on HLA, their predictive value alone is still limited. Tools available for proteasomal peptide processing have been trained with a combination of data sets derived from *in vitro* digestion assays with the conventional proteasome, and naturally processed HLA-I ligands, which also include those processed by the immunoproteasome (44, 45). Likewise, peptide transport prediction algorithms have been trained with data sets of experimentally validated HLA-I peptides known to bind TAP (46, 47). However, TAP-independent processing pathways also contribute to the peptide repertoire, and these cannot be predicted with currently available transport prediction tools (48). Given the yet limited predictive value of these *in silico* prediction tools, they are typically integrated with more robust predictors in pipelines for neoantigen prioritization (49, 50).

Algorithms capable of predicting peptide binding to HLA molecules are the most widely used for *in silico* prioritization of neoantigens and were instrumental for the first identification of neoantigens using tumor WES (7, 51). These tools are usually trained with large datasets of experimentally defined HLA ligands and peptides eluted from HLA molecules using mass spectrometry (MS)-based immunopeptidomics. Peptide HLA binding prediction takes into account not only the importance of anchor residues but also the influence of amino acids flanking them. Additionally, the diversity of HLA molecules, which gives rise to thousands of alleles with distinct binding preferences, are considered. Since generating experimental data for that amount of alleles is not feasible, prediction tools used to date incorporate biochemical and structural data of known alleles to infer peptide binding to rare alleles for which no or little data is available (52).

In order to predict which mutated peptides are more likely to bind to HLA, binding affinity prediction tools are commonly fed with a list of peptides in which the detected mutation is flanked by a variable number of amino acids of the wild-type sequence. Algorithms then generate small peptides (8–14 amino acids) from the input sequence for which the binding affinity to the queried HLAs is predicted. Since several peptides derived from the same 25mer sequence are likely to bind one or more HLAs, the potential number of candidate peptides can sometimes increase (Figure 1). Given that *in vitro* neoantigen screening assays currently limit the number of tested peptides to the hundreds, peptides are commonly prioritized based on binding affinity ranking. Predictors such as NetMHCpan 4.0 generally report results as either IC50 values in nM units or as a percentile rank score. IC50 values reflect direct binding affinity predictions, and thresholds <500 nM can be used to define candidate peptides that are more likely to bind to HLA. Percentile rank scores reflect relative binding affinity to a specific HLA allele compared to a large set of random peptides, and ranks ≤ 2 are used as thresholds for selecting potential neoantigen binders (53). Although both outputs can be used, the percentile rank is preferred to select candidate peptides across multiple HLA molecules, as it is less influenced by the large differences in peptide binding affinity values among HLA molecules.

Although prediction tools for HLA-II-restricted peptides also exist, these are less reliable than HLA-I predictors for two main reasons. First, endosomal HLA-II peptide processing is complex and poorly characterized (54), limiting the development of HLA-II peptide processing algorithms. Second, prediction of binding affinity to HLA-II molecules is more complex due to its structural nature because, unlike HLA-I molecules in which the peptide-binding groove is closed, HLA-II molecules have open ends. Even though the core binding motif of both molecules comprises peptides of approximately nine amino acids, HLA-II-restricted ones have a wider length range (11–20 amino acids) compared to HLA-I-restricted ones (8–11 amino acids), and the flanking amino acids can affect binding affinity (55). Further research addressing these challenges will be crucial for improvement of HLA-II prediction tools in the future.

Despite advances in prediction algorithms, currently available tools fail to reliably predict which of the presented peptides will be immunogenic (i.e., whether a presented peptide will be recognized by T cells). This is one of the main limiting steps in neoantigen screening, and it is perfectly reflected by the fact that only few of the hundreds of peptides identified by tumor WES data and *in silico* prediction are immunogenic despite binding to HLA molecules. Although HLA binding prediction is a strong correlate of immunogenicity, accumulating data suggest that bias of *in silico* prediction toward strong binders (<500 nM) can overlook immunogenic peptides that show low-binding affinity. The first evidence of this was reported by Duan et al., who developed an algorithm, termed differential agretopicity index (DAI), which ranks mutant peptides based on their improved binding to HLA compared to the wild-type counterpart (34). Using DAI to identify neoantigens in mouse models of cancer, the study demonstrated that validated immunogenic peptides could have binding affinities up to 140-fold higher than the 500 nM threshold. These findings have been confirmed by other studies in humans (17, 18, 28, 56), highlighting that peptide selection based on the 500 nM threshold should be revisited. Additionally, other limitations of binding prediction tools have been recently identified in clinical trials of cancer vaccines. Patients with melanoma or glioblastoma receiving personalized neoantigen vaccines appear to favor CD4^+^ over CD8^+^ T-cell responses against the immunizing peptides, even though these were predicted and prioritized using HLA-I binding algorithms (21, 22). These data further stress the need of developing improved algorithms which can reliably predict HLA-I and II immunogenic peptides. The low number of immunogenic neoantigens validated to date [ <300; reviewed in Karpanen and Olweus (32)] makes it difficult to generate a consensus for features likely to predict peptide immunogenicity. Although this is currently a matter of extensive research, only few parameters, besides the aforementioned DAI, have been suggested to improve the prediction of immunogenicity of peptides. For instance, differences in non-anchor residues (P4-P6), peptide size (i.e., large) and amino acid composition (i.e., aromatic residues) have been associated with immunogenicity (57). Additionally, peptide-HLA (pHLA) stability, measured by biochemical assays, has been proposed as a parameter to discriminate immunogenic from non-immunogenic peptides (58). Data derived from this kind of experiments led to the development of prediction tools which show that immunogenic peptides promote more stable pHLA-I than non-immunogenic peptides (59). However, the predictive value of this tool is still controversial (60). Thus, its use as a single predictor is less frequent.

It is worth mentioning that the immunogenicity of a given neoantigen does not necessarily translate into tumor rejection and/or therapeutic benefit. Mice studies have shown that the vast majority of identified neoepitopes, despite triggering T-cell responses, fail to induce complete tumor rejections (34, 35). In humans, the previously mentioned clinical trials of personalized neoantigen vaccines have rarely shown clinical responses despite triggering strong T-cell responses against the targeted neoantigens (20–23). The development of novel algorithms or screening tools capable of identifying neoantigens capable of inducing tumor rejection could be determinant for the efficacy of personalized vaccines.

To date, there is a plethora of computational pipelines that allow the identification of NSM and the prediction of neoantigens. However, these are built on the basis of traits set by each developer, which often leads to discordant results. Strategies such as the Tumor Neoantigen Selection Alliance (TESLA), which seek to harmonize these pipelines, will be of great importance in the coming years to improve neoantigen identification (61).

#### Selection of Candidate Neoepitopes Using Mass Spectrometry-Based Immunopeptidomics

Another possible strategy that can be used to prioritize candidate neoantigens for screening is the use of MS-based immunopeptidomics which relies on the study of the tumor pHLA immunopeptidome (62). This method starts with the lysis and homogenization of the tumor material followed by the purification of the pHLA complexes through immunoprecipitation. After eluting the peptides bound to HLA molecules, liquid chromatography coupled tandem MS (LC-MS/MS) is performed to identify the amino acid sequence of the eluted peptides, which is commonly obtained by matching MS/MS spectra against a customized protein sequence database (63). This database is generated by combining a reference protein sequence database with genomic information derived from patient's NGS data, which is essential to identify eluted mutated peptides that are private for each patient. This method to identify neoantigens was first described in a mouse tumor model. WES and RNAseq in combination with MS analysis of peptides eluted from the cell surface MHC of two mouse tumor cell lines allowed the identification of seven candidate neoantigens, three of which turned out to be truly immunogenic (64). Since then, candidate neoantigens have also been successfully identified in human tumor cell lines (65) and, more importantly, in fresh tumor material (56, 66). Indeed, Bassani-Sternberg et al. demonstrated for the first time that this strategy could also be exploited to identify immunogenic neoantigens directly from primary human cancer tissues. In this case, the combination of WES and immunopeptidomics of tumors from five patients allowed the identification of 11 mutated peptides, and two of eight peptides tested were able to elicit antitumor T-cell responses.

MS-based immunopeptidomics is advantageous, as it substantially narrows down the list of candidate neoantigens to be screened (Figure 1) and, consequently, the number of false positives that are obtained using other strategies such as *in silico* prediction (67). This might be of great importance for immunogenicity screening assays, especially in tumors with high mutation burden. Additionally, this is currently the only unbiased method that directly interrogates the naturally presented HLA-bound peptides including those harboring post-translational modifications (68). Neoantigens could also derive from non-canonical or cryptic peptides, including those derived from alternative open reading frames, novel exon-exon junctions, intronic sequences, long non-coding RNAs, 5′ untranslated regions (5′UTRS; Table 2). These could also be identified by performing database-dependent analyses as long as the amino acid sequences of such peptides have been previously introduced into the customized protein sequence database (80, 81). This could be achieved using a customized database derived from RNAseq data as exemplified by the study of Smart et al., in which they identified epitopes derived from retained introns using RNAseq and validated their expression and presentation by MS analyses (42). Importantly, retained introns expressed in normal tissues were filtered out with the aim to exclusively identify those that are tumor-specific and can potentially be immunogenic. As an alternative to the generation of a customized sequence database, the amino acid sequence can also be directly extracted from tandem mass spectra through database-independent analysis (i.e., *de novo* sequencing). However, the use of this strategy is still limited because it is error prone and fails to determine the entire amino acid sequences due to incomplete tandem mass spectra (82).

**Table 2:** Tumor-rejection antigens derived from non-canonical protein sequences.

**Epitope identified^a^**	**Type of tumor**	**Origin of non-canonical peptide**	**Expression in normal tissue**	**Gene name**	**HLA restriction element**	**Reference**
MSLQRQFLR	Melanoma	aORF	Unknown	*TRP1*	HLA-A^*^31	(69)
VYFFLPDHL	Melanoma	Intronic	Yes	*GP100*	HLA-A^*^24	(70)
RSDSGQQARY	Melanoma	Intronic	Yes/low^b^	*AIM2*	HLA-A^*^01	(71)
VLPDVFIRC/VLPDVFIRCV	Melanoma	Intronic	No	*GNTV*	HLA-A^*^02:01	(72)
EEKLIVVLF	Melanoma	Intronic	No	*MUM1*	HLA-B^*^44:02	(4)
LPAVVGLSPGEQEY	Renal cell carcinoma	aORF	Yes	*MCSF*	HLA-B^*^35:01	(73)
SPRWWPTCL	Renal cell carcinoma	aORF	Yes/low^b^	*iCE*	HLA-B^*^07:02	(74)
EVISCKLIKR	Melanoma	Intronic	No	*TRP2*	HLA-A^*^68:011/HLA-A^*^33:01	(75)
LAAQERRVPR	Melanoma and breast cancer	aORF	Unknown	*NYESO1*	HLA-A^*^31	(76)
MLMAQEALAFL	Melanoma	aORF	Yes	*LAGE1*	HLA-A^*^02:01	(77)
CQWGRLWQL/MCQWGRLWQL	Melanoma	aORF	Unknown	*BING4*	HLA-A^*^02	(78)
LPRWPPPQL	Renal cell carcinoma	Intronic	Yes	*RU2*	HLA-B^*^07	(79)

a identified by cDNA library screens;

b *compared to cancer tissue; aORF, alternative open reading frame; HLA, human leukocyte antigen*.

Although MS-based immunopeptidomics offers multiple advantages, the discovery of presented immunogenic peptides using this approach is hindered by technical limitations, evidenced by the short list of human cancer neoepitopes identified through this approach to date (56, 66). The major concern is the low sensitivity of MS. The fact that MS is skewed toward detecting the more abundant peptides hampers the identification of mutated peptides among all endogenously presented peptides, especially if they are expressed at low levels or exclusively expressed in subclonal tumor populations. Because of this, and considering that tumor cells express heterogeneous levels of HLA molecules, large amounts of starting tumor material is required to identify candidate neoepitopes. Indeed, in the study by Bassani-Sternberg et al. in which they eluted HLA-I and II bound peptides from primary tumor material, tumor biopsy size seems to be associated with the number of mutated peptides detected (66). Identifying candidate neoantigens within the repertoire of HLA II-peptides in fresh tumor material can also be cumbersome probably due to their low frequency within the pool of presented peptides on APCs, which typically express HLA-II molecules. In fact, even if HLA-II peptides have been successfully eluted in different studies, neoantigens have not been identified so far among the class II tumor peptidomes (67, 83).

Another important consideration is that MS/MS relies on efficient ionization and fragmentation of the peptides. Thus, the successful identification of the sequence of a peptide will depend on its amino acid composition and the biochemical characteristics of such amino acids, which will determine their capacity to be ionized and efficiently fragmented (84). Consequently, a fraction of peptides that are naturally presented might never be detected using this approach.

Overall, this strategy yields a long list of minimal epitopes from both normal and mutated HLA-bound peptides, from which candidate neoantigens can be selected and tested to assess their immunogenicity.

#### Unfiltered Neoantigen Candidate List

Once all tumor NSM are identified, one possibility is to interrogate the immunogenicity of all candidate neoepitopes identified by tumor WES, without biasing the selection of peptides based on *in silico* prediction, which may not always be accurate. This can be done using a variety of immunological screening methods, as explained in section Immunological Screening Methods Used to Evaluate Neoantigen Recognition. However, the feasibility of this approach is restricted to tumors with a limited number of mutations given the cost and effort associated with screening T cells for recognition of a large set of mutated epitopes. Alternatively, and particularly when dealing with tumors with high mutation burden, it is crucial to further filter candidate neoantigens to exclusively evaluate the immunogenicity of a selected set of candidate neoantigens.

## Evaluation of Immunogenicity of Candidate Neoantigens

Evidence arising from available studies is that the vast majority of selected candidate neoantigens identified in a tumor are not recognized by T cells (28–30, 32, 85). Thus, evaluation of the immunogenicity of candidate neoantigens using a variety of screening methods will be critical to more precisely identify and select neoantigens suitable for clinical intervention (Table 3).

**Table 3:** Immunological screening assays used to test for neoantigen recognition.

**Strategy**	**Advantages**	**Disadvantages**
cDNA libraries	Interrogates all transcribed sequences	Labor intensive and time-consuming. Biased toward highly transcribed genes. Influenced by the size, expression levels or GC-richness of transcripts encoding for T-cell epitopes. Interrogates mutated and non-mutated sequences.
Minimal epitopes	Cost-effective. HLA-matched target cells (based on *in silico* prediction) can be used instead of autologous APCs	Exclusively interrogates a selected list of mutated epitopes based on *in silico* prediction or validated by immunopeptidomics Requires autologous or HLA-matched cells as target cells Not optimal for CD4^+^ cells
Peptide-HLA multimers	Overcomes the need of autologous APCs Allows the isolation of antigen-specific T cells	Exclusively interrogates a selected list of mutated epitopes based on *in silico* prediction or validated by immunopeptidomics Multimers are available for a limited number of HLA molecules Not optimal for CD4^+^ cells
Tandem minigenes or peptide pools	Can be used to interrogate all or a large portion of mutated epitopes Allows potential processing and presentation of candidate neoantigens on HLA-I and HLA-II Does not require prior knowledge of the minimal epitope or HLA restriction	Cost increases in patients with high mutation burden Availability of APCs/effectors can limit this approach, especially when >250 epitopes are tested Requires autologous APCs as target cells Peptide processing by immunoproteasome in APCs might differ from processing by the proteasome in tumor cells

*APC, antigen-presenting cell; HLA, human leukocyte antigen*.

### Immunological Screening Methods Used to Evaluate Neoantigen Recognition

The first strategy employed to identify human T-cell reactivities to neoantigens was described in Coulie et al. (4). Coulie et al. identified a tumor-specific intronic mutation in MUM-1 recognized by a human cytolytic T lymphocyte (CTL) clone using an approach which involved screening melanoma-specific CTLs for recognition of target cells transfected with tumor cDNA library pools along with the appropriate HLA restriction element. Additional mutated gene products derived from CDK4 and β-catenin, capable of inducing T-cell responses, were also identified using similar strategies and were found to either enable peptide binding to HLA-I by creating an HLA-I binding motif or to modify a TCR contact residue of a peptide that was already capable of binding to HLA-I (9, 86). This strategy was widely used during the following decades to dissect the molecular nature of antigens recognized by tumor-reactive T cells, leading to the identification of additional neoantigens (10, 11, 87). However, this approach is laborious and time-consuming, it can be influenced by the size, expression levels or GC-richness of transcripts encoding for T-cell epitopes, and optimally requires the establishment of tumor-specific clones and matched tumor cell lines, which is often not possible. Furthermore, this approach unbiasedly screens T cells for recognition of both mutated and non-mutated antigens, leading to frequent identification of self-antigens, rather than neoantigens (Table 3).

All these limitations have incentivized the development of alternative high-throughput immunological strategies that facilitate the evaluation of T-cell reactivity against a large number of candidate neoepitopes identified by tumor WES. Yet, it is worth mentioning that a considerable number of tumor-rejection antigens identified by screening tumor cDNA libraries, including the first human neoantigen identified (4), derive from non-canonical protein sequences encoded by introns, alternative open reading frames or aberrantly spliced variants (Table 2). These findings are of potential concern, given that the current strategies exclusively identify NSM in exons (rarely using WGS), ignoring potential neoantigens that could arise from non-canonically translated sequences. Current efforts to overcome this limitation of exome-based strategies to identify neoantigens arising from non-canonical protein sequences combine WES with RNAseq and immunopeptidomics, as previously explained in detail in section Selection of Candidate Neoepitopes Using Mass Spectrometry-Based Immunopeptidomics.

#### Screening of Predicted or Eluted Minimal Neoepitopes

In 2012, two reports in mouse tumor models demonstrated for the first time that tumor WES can be exploited to identify neoantigens (35, 88). In 2013, Robbins et al. performed a retrospective study to identify the molecular nature of the antigens targeted by TILs from five melanoma patients, some of which demonstrated tumor regression following TIL transfer (7). They used tumor exome sequencing to identify all NSM and synthesized neoepitopes that were predicted to bind to the patients' HLA-A class I molecules and screened the TIL infusion products for recognition of the mutated peptides individually pulsed onto COS7 monkey kidney cells or HEK293 human embryonal kidney cells transfected with the appropriate HLA-A alleles. This work led to the identification of eight mutated peptides recognized in four of five patients analyzed. Remarkably, two of the neoantigens that were identified in two independent patients using this approach, CSNK1A1 and PLEKHM2, were not identified using the tumor cDNA screening method. This work describing frequent detection of neoantigen-specific lymphocytes in responding patients together with a recent study demonstrating that patients that exhibited complete tumor regressions following tumor-infiltrating lymphocyte (TIL) therapy (6) have a higher tumor mutation burden suggest that neoantigen-specific lymphocytes play an important role in the efficacy of TIL therapy.

Although this screening strategy was initially used to interrogate reactivity to neoepitopes presented exclusively by HLA-A alleles, it can be used to identify neoantigens in any HLA of interest as long as autologous or HLA-matched antigen-negative target cells are available or by introducing the autologous HLA molecules into transfectable cells, that can be used as target cells. It can also be used to interrogate candidate neoepitopes eluted from the cell surface HLA molecules of tumor cell lines or tumor biopsies (65, 89). In a slightly higher-throughput version, it can be used to interrogate large numbers of *in silico* predicted neoepitopes by grouping these into peptide pools. It is the simplest approach available to analyze neoantigen immunogenicity, since it relies on classically available immunological techniques such as IFN-γ release by ELISA or ELISPOT assays, as well as others, and its sensitivity depends on the specific read-out chosen to measure T-cell responses. This approach has allowed to successfully identify immunogenic neo-epitopes in different malignancies including melanoma, NSCLC and ovarian cancer (56, 65, 90, 91).

A second immunological method that can be used to identify neoantigen-specific lymphocytes is the use of pHLA multimers. Since pHLA-I tetramers were described in 1996, these have become essential reagents for the visualization and isolation of antigen-specific T cells (92). However, the technically challenging generation of individual pHLA monomers coupled with the limited number of fluorochromes available for pHLA multimer detection precluded a more comprehensive analysis of T-cell immunity. Two technical innovations have contributed to facilitate large scale neoepitope discovery using HLA multimer-based detection technologies from limited biological material. First, the development of conditional HLA ligands which are cleaved upon exposure to UV-light and can be exchanged with any epitope of interest (93, 94). Using UV-exchangeable HLA ligands, only one pHLA multimer loaded with an exchangeable peptide has to be produced for each HLA allele of interest and can be used as a stock to generate large libraries of pHLA complexes through simple manipulations. Similar strategies have been reported recently, all of them aiming at facilitating the high-throughput production of large panels of pHLA complexes (95, 96). Second, fluorochrome-based combinatorial encoding has increased the number of T-cell specificities that can be interrogated by flow cytometry in one sample (up to 28 single specificities with two-dimensional combinatorial encoding with eight fluorochromes) (97). In one study, Van Rooij et al. performed tumor WES, and expanded TILs from a melanoma patient who exhibited a partial response to ipilimumab. They used *in silico* HLA-A and HLA-B binding prediction algorithms to identify neoepitope candidates and generated a library of pHLA tetramers. TILs were screened for binding to this library of tetramers using the fluorochrome-based combinatorial encoding staining method and this led to the identification of TILs targeting two distinct neoantigens (51). Interestingly, they also monitored an increase in the frequency of one of the neoantigen-specific lymphocytes in the blood of the patient following treatment with anti-CTLA-4, suggesting the involvement of these T cells in the therapeutic efficacy of this immunotherapy.

This technology has enabled the generation of large panels of desired pHLA complexes and consequently pHLA multimer libraries are currently used for large-scale immunogenic neo-epitope discovery (98), and have successfully been used to identify immunogenic mutated neoepitopes in NSCLC and melanoma (14, 99). More recently, DNA barcoding of individual pHLA molecules has enabled to screen 1031 T-cell specificities in one single reaction (100). While DNA barcodes offer the possibility of screening T cells for a full cancer mutanome using one biological sample, this technology only provides a measure of T-cell frequency, but lacks the visual assessment of the individual T-cell reactivities as well as the possibility of performing short-term culture given that T cells are lysed for DNA barcoding amplification.

High-throughput screening of T cells using multiplexed pHLA multimer staining is of particular interest as it overcomes the need of autologous or HLA-matched APCs. However, pHLA complexes are only available for a limited number of HLA allotypes. Thus, if the aim is to screen T cells for recognition of all possible predicted or HLA-eluted neoepitopes, this strategy can only be used in patients for which all or most of the HLA allotypes are available for pHLA multimer generation. The detection of CD4^+^ specificities using HLA-II multimers represents an additional challenge in the field. Although it is feasible (101), the low accuracy of *in silico* prediction of HLA-II-restricted epitopes can result in a less precise identification of candidate minimal epitopes (see section Selection of Candidate Neoantigens using *in silico* Peptide Prediction). Furthermore, technical issues related with the production of pHLA-II multimers, and the weaker TCR binding affinities to HLA-II also hinder the use of pHLA-II multimer staining for neoantigen-specific CD4^+^ T-cell identification (102). Consequently, the majority of screenings performed using this approach are usually focused on identifying neoepitopes presented on HLA-I molecules to CD8^+^ T cells, which might underestimate the contribution of neoantigen-specific CD4^+^ T cell populations.

It is worth mentioning that the immunological functional screening assay as well as the HLA multimer staining technologies described above rely on in-house or commercial production of synthetic peptides. These are frequently synthesized or ordered at <70% purity, given the relatively large number of neoepitopes obtained following *in silico* peptide prediction algorithms and the costs associated with custom peptide production. However, custom peptide libraries have been reported to contain impurities, that can affect T-cell recognition and yield false-positive results (103, 104). Hence, validation of neoantigen-specific reactivity/ies using a second batch of >70% pure peptides is highly advisable. Ultimately, the best *in vitro* evidence that a neoantigen exists is provided by showing preferential T-cell recognition of a given neoantigen expressed, processed and presented by autologous APCs or HLA transfectable target cells, compared to the corresponding wild-type (wt) counterpart.

#### Unbiased Screening of All Candidate Neoantigens Identified by Tumor WES

While the strategies mentioned above are frequently used to identify neoantigens and neoantigen-specific lymphocytes, they are limited by the accuracy of current *in silico* prediction algorithms, which have not been thoroughly trained to identify minimal epitopes for rare HLA-I alleles or HLA-II molecules, and do not consider post-translational modifications (see section Selection of Candidate Neoantigens using *in silico* Peptide Prediction). To overcome these limitations, Lu et al. devised a new screening assay to evaluate CD8^+^ and CD4^+^ T-cell responses to any of the NSM identified expressed processed and presented on the patient-specific HLA-I and HLA-II molecules, without the need for *in silico* prediction. Briefly, for each NSM identified one minigene construct was designed, encoding the mutated amino acid flanked by 12 amino acids of the wt sequence. Typically, between 6 and 24 minigenes were stringed together to generate tandem minigenes (TMG) in a single open reading frame. *In vitro* transcribed RNA generated from the TMGs was transfected into autologous APCs, such as B cells or immature dendritic cells (8, 25, 29). In addition, or as an alternative to the generation of mutated TMGs, 25-residue peptides can be synthesized and grouped into peptide pools (PPs), each containing up to 24 mutated peptides. Neoepitopes presented through intracellular (transfected TMGs) and extracellular (pulsed peptides) pathways on autologous APCs expressing all HLA-I and HLA-II molecules are then evaluated for their ability to induce T-cell responses and when reactivities are detected against a specific TMG or PP, these are subsequently deconvoluted to identify the specific neoantigen recognized.

This unbiased screening approach was used to identify two mutated antigens, KIF2C and POLA2, targeted by TIL derived from two patients that underwent complete tumor regression following TIL transfer (8). An additional study interrogated the immunogenicity of 720 non-synonymous somatic variants identified by WES, encoded by 62 TMGs, and identified 10 neoantigens targeted by TILs presented on three different HLA molecules (33). Linnemann et al. used immortalized autologous B cells pulsed with 31-residue mutated peptides to identify neoantigen-specific CD4^+^ T cells in two of three melanoma patients evaluated (85). Moreover, this high-throughput screening approach revealed that neoantigen-specific lymphocytes are frequently detected in TILs derived from GI cancers which have lower mutation burden (29), providing an opportunity to develop effective immunotherapies for patients with additional cancer types. Importantly, TMG and/or PP screening were used to prospectively select neoantigen-specific TILs for patient treatment and this was able to induce antitumor responses in a patient with cholangiocarcinoma treated with CD4^+^ ERBB2IP mutation-specific lymphocytes (25), a patient with metastatic colorectal cancer treated with CD8^+^ KRAS mutation-specific lymphocytes (26), and a patient with breast cancer treated with TILs recognizing SLC3A2, KIAA0368, CADPS2, and CTSB mutated gene products combined with anti-PD-1 (27). A diverse repertoire of lymphocytes targeting three neoantigens was also detected using this approach in one patient with cervical carcinoma who underwent complete tumor regression following TIL transfer (105). Overall, this strategy has been used to identify over 100 neoantigens in over 13 studies (8, 25–31, 33, 105–108), including both CD4^+^ and CD8^+^ T-cell responses. Moreover, this provides the strongest evidence that T-cell therapies targeting neoantigens can lead to antitumor responses. However, these unbiased screening strategies have also provided evidence that only a limited number of tumor somatic mutations detected by tumor WES and RNAseq are immunogenic.

The biggest advantage of the unbiased screening using TMGs and PPs is that it mimics the natural antigen processing and presentation of neoepitopes on both class I and class II patients own HLA molecules, overcoming the need of *in silico* peptide prediction algorithms. Importantly, it has enabled the identification of CD4^+^ neoantigen-specific lymphocytes. However, it also has some limitations that should be taken into account. Although, theoretically, all mutations identified by WES can be screened, the cost associated with peptide and TMG synthesis, and *in vitro* RNA transcription greatly increases when screening tumors with high mutation load. Moreover, since the initial screening is carried out with TMGs and PPs containing multiple candidate neoantigens, a deconvolution is required to identify the neoantigen recognized. Thus, the availability of large numbers of autologous APCs and effector cells to assess the immunogenicity of neoantigens can sometimes be a limitation. The lack of autologous APCs could be overcome using HLA-matched cells or by transfecting the individual HLA alleles, although this further complicates the screening strategy. In addition, the electroporation of TMG RNA does not guarantee expression and processing of all the mutated minigenes included, which could be influenced by the position in the TMG or the 3D structure of the chimeric protein resulting from the concatenation of up to 24 minigenes. Moreover, the size of the mutated minigene or peptide to ensure proper processing and presentation is still a matter of debate. Finally, the efficiency of this approach is influenced by the APC chosen. Although immature dendritic cells and *ex vivo* stimulated B cells are the cells of preference, their proteasome can be different to that expressed in tumor cells, and the ability of each cell type to process and present TMG or cross-present peptides could differ.

In conclusion, this strategy allows to agnostically interrogate the immunogenicity of all or a large fraction of candidate neoantigens detected in a given tumor without prior knowledge of the minimal epitope or the HLA restriction element of each mutated peptide.

#### Novel High-Throughput Screening Strategies to Identify Neoantigens

A few novel technologies that have recently been described aim at identifying the cognate peptide recognized by a T cell through the detection of APCs that have been specifically recognized by T cells, rather than monitoring specific activation of T cells. For instance, Joglekar et al., developed a cell-based platform for T-cell antigen discovery that relies on the screening of a large number of antigens through the expression of chimeric receptors termed *Signaling and antigen-presenting bifunctional receptors* (SABRs) in NFAT-GFP-Jurkat cells through stable transduction with lentiviral vectors (109). These chimeric receptors are composed of an extracellular domain comprising a peptide tethered to an HLA fused to an intracellular CD3ζ signaling domain and a CD28 co-stimulatory domain. When recognized by a specific TCR, this interaction triggers the expression of GFP and CD69 on NFAT-GFP-Jurkat cells which can be selected and sequenced to identify the specific peptide recognized. More recently, Kisielow et al. have used a similar NFAT reporter system which is restricted to the identification of tumor-specific peptides recognized by CD4^+^ T cells (110). In this case, the signal-triggering molecule is a MHC-TCR chimeric receptor (MCRs) which incorporates the peptide linked to the MHC domain. MCR libraries are generated by cloning fragmented tumor cell cDNA into MCR sequences and are transduced into reporter cells, which are used as target cells in co-culture assays with T cells encoding for TCR of interest. TCR interaction with a specific MCR induces reporter gene expression through NFAT activation, allowing the selection, and identification of the recognized peptide through sequencing. Although these are proof-of-concept studies and their applicability as well as advantages and limitations remain to be determined, the novel strategies described may potentially be used alone or in combination with other screening strategies for an unbiased identification of neoantigens targeted by T cells in patients with cancer.

### Sources of Effector T-Cell Populations to Identify Neoantigen-Specific Lymphocytes

**Figure 2:**
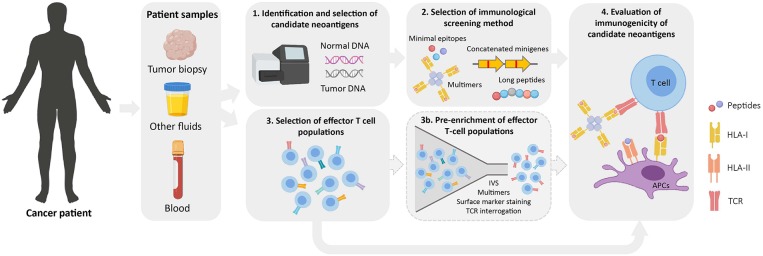
Workflow for the identification of immunogenic neoantigens. Normal and tumor DNA obtained from cancer patients are used to identify tumor-specific NSM by WES (1). Upon selection of candidate neoantigens (see Figure 1), different immunological screening methods can be used to evaluate peptide immunogenicity (2). Prior to evaluating their immunogenicity, effector T-cell populations of interest are selected from cancer patient samples (3). These can either be directly used in immunological screening assays or as a starting source for an optional pre-enrichment step to increase the frequency of neoantigen-specific T cells (3b). Finally, the immunogenicity of candidate neoantigens is evaluated using the screening method and effector T-cell population/s of choice (4). IVS, *in vitro* sensitization; APC, antigen-presenting cell; TCR, T-cell receptor.

Once a list of candidate neoantigens is obtained, their immunogenicity is typically evaluated *in vitro*. In addition to the immunological screening methods previously described, the selection of an effector T-cell population with which screening assays will be performed is a critical determinant for neoantigen identification (Figure 2). Theoretically, any tissue or fluid from which T cells can be isolated and/or expanded is a potential source for neoantigen immunogenicity screenings.

#### TILs and Other Tumor-Associated Populations

T cells are thought to accumulate at the tumor site, presumably as a result of local antigen-specific clonal expansion. Consistent with this, the tumor-infiltrating TCR repertoire is typically more oligoclonal as shown by intratumoral TCR deep sequencing (111, 112). It is therefore not surprising that TILs are the preferred T-cell source to detect T cells recognizing neoantigens. The optimization of TIL culture conditions in the late 1980s (113), motivated in part by the therapeutic potential of adoptive cell transfer (ACT), has facilitated the expansion of the relatively small numbers of lymphocytes that can be naturally found infiltrating human tumors. TIL cultures, which are usually expanded from tumor biopsies in the presence of high IL-2 concentrations, have been used to identify immunogenic HLA-I- and HLA-II-restricted neoantigens (7, 25, 29, 51, 111, 114). Despite being the most attractive source in terms of T-cell composition, expansion of TILs is not always successful, it can be highly heterogeneous even when expanding TILs from contiguous tumor fragments, and the generation of these cultures depends on tumor biopsies which are not always available. Furthermore, *in vitro* expansion of TILs can significantly increase or decrease the frequency of antigen-specific T cells (99, 115), thereby underestimating the initial T-cell repertoire. Recent studies have also shown that TILs are composed not only of tumor-reactive but also of cancer-unrelated T cells (e.g., virus-specific T cells) (116, 117). How these bystander cancer-unrelated T cells behave in comparison to tumor-reactive cells during TIL expansion has not been fully determined, although initial studies suggest that *ex vivo* expansion of TILs can increase the frequency of virus-specific T cells at the expense of tumor-reactive T cells (115). Therefore, other T-cell sources have been studied with the aim of complementing TILs for neoantigen validation.

Fluids directly associated with particular solid tumors, such as ascites from ovarian cancer or pleural effusions from mesotheliomas, have been used as sources for the expansion of tumor-associated lymphocytes (TALs). TALs do not fully share TCR repertoires with TILs (118), and they might thus underrepresent the tumor-reactive T-cell population of the primary tumor. Nonetheless, the potential of TALs has been demonstrated in a high-grade serous ovarian cancer patient in which a neoantigen-specific T-cell clone was detected in ascites at the time of recurrence, but not in primary ascites or tumor samples (90). Other body fluids, such as cerebroespinal fluid (CSF), although low or absent in healthy individuals, can be increased in patients with different pathologies (119). Indeed, T cells isolated and expanded from CSF of patients with diffuse intrinsic pontine glioma have been used to detect tumor-reactive T cells after dendritic-cell vaccination (120). Urine has been recently used to isolate and characterize lymphocytes from bladder cancer patients (121). Notably, urine-derived lymphocytes (UDLs) recapitulated the phenotypic and TCR landscapes of T cells from the tumor microenvironment. Given the non-invasive nature of urine collection and the similarities between UDLs and TILs, the former represents an attractive source of T cells to identify immunogenic neoantigens in bladder cancer patients.

#### PBMCs

One of the major challenges for the identification of neoantigens is finding non-invasive T-cell sources to perform immunological screenings. PBMCs derived from blood extractions represent the most attractive source for this purpose. The first evidence of circulating neoantigen-specific T cells was reported more than 20 years ago. Back then, effector T cells used for reactivity screenings were obtained using a mixed lymphocyte tumor culture (MLTC) (122). After successive rounds of stimulation of PBMCs from cancer patients with irradiated autologous tumor cell lines, they were tested for tumor reactivity, and positive “clones” were obtained. Using MLTC-derived clones, Wölfel et al. identified an HLA-A2.1-restricted neoepitope derived from CDK4 (86). This strategy has been successfully exploited in other studies, but the requirement of multiple stimulations prompted the development of strategies to detect neoantigen-specific lymphocytes in unmanipulated PBMCs. The first reports of circulating neoantigen-specific T cells detected in bulk PBMCs from cancer patients are from less than a decade ago with the advent of improved multimer staining technologies (51, 99). In one of those studies, Cohen et al. used multimer libraries to screen for candidate neoantigens, resulting in the successful isolation and expansion of neoantigen-specific T cells from the blood of melanoma patients. Subsequent studies have also shown that naïve T cells from healthy donors can be used as a source to identify neoantigens from melanoma patients for which T-cell reactive clones were absent in autologous TILs (123). These studies suggest that blood-derived T cells are attractive populations for identifying immunogenic neoantigens.

#### Pre-enriched T-Cell Populations

Multimer-based studies have shown that neoantigen-specific T cells are present at relatively low frequencies in fresh tumor single-cell suspensions (98), and this is even more problematic when working with peripheral blood lymphocytes (PBLs) (51, 99). To overcome this challenge, different enrichment strategies have been developed to increase the odds of detecting cells which otherwise would be missed due to limited technical sensitivities. Given the low frequency of neoantigen-specific T cells, enrichment strategies rely on the selection of particular T-cell populations that are then *in vitro* expanded to achieve high cell numbers for immunogenicity screenings (Figure 2; Table 4).

**Table 4:** Strategies to enrich for neoantigen-specific lymphocytes.

**Strategy**	**Advantages**	**Disadvantages**
Surface marker-based selection	Prior knowledge of the specific reactivity or HLA restriction is not required Universal (Can be used for every patient) Increases the frequency of neoantigen-specific lymphocytes	Expression of surface markers varies among patients May not capture all reactivities Does not exclusively select neoantigen-reactive lymphocytes
Multimer staining	Allows isolation of T cells with one specific reactivity with high purity	Requires generation of HLA multimer for each reactivity Limited number of HLA multimers Prior knowledge of the specific reactivity and HLA restriction required Not optimal for isolating tumor-reactive CD4^+^ T cells Sensitivity limited by the frequency of the neoantigen-specific population
*In vitro* sensitization	Increases the frequency of T cells with a specific reactivity	Requires multiple rounds of *in vitro* sensitization Requires autologous or HLA matched APCs Laborious depending on the number of peptides screened

*APC, antigen-presenting cell; TCR, T-cell receptor; HLA, human leukocyte antigen*.

One of these strategies exploits the fact that, upon recognition of their target antigen on tumor cells, T cells express co-inhibitory and co-stimulatory molecules. Furthermore, chronic exposure to target antigens may differentiate TILs into a dysfunctional (also termed exhausted) state characterized by the co-expression of exhaustion/activation markers (124, 125). This T-cell phenotype has prompted research evaluating whether the expression of these markers could be used to identify and enrich for neoantigen-specific T-cells residing in fresh tumors or peripheral blood of patients with cancer. To date, most of the co-inhibitory/co-stimulatory markers identified to associate with enrichment of tumor- or neoantigen-reactive T cells have been described in TILs from fresh tumor preparations. Initial reports demonstrated that the isolation and expansion of CD8^+^ melanoma TILs based on either PD-1, or a combination of PD-1 TIM-3 and LAG-3 expression consistently enriched for T cells recognizing tumors and neoantigens (111, 126). Subsequent studies have confirmed that tumor-specific CD8^+^PD-1^+/hi^-infiltrating populations show a distinct transcriptional and metabolic profile (127). Phenotypic characterization of CD8^+^ TILs from colorectal and lung cancer patients has revealed that CD39, rather than PD-1, could accurately distinguish between tumor-specific (CD39^+^) and cancer-unrelated T cells (CD39^−^) (117). In line with this, a recent study has shown that co-expression of CD39 and CD103 favors the identification of tumor-reactive T cells (128). A different approach exploits the fact that T cells express CD137 upon recognition of tumor cells (129). Consequently, isolating T cells based on CD137 expression after co-culture with autologous tumor cells leads to enrichment of neoantigen-specific T cells (130, 131). Identification of markers associated with neoantigen-specific T-cell enrichment in circulating T cells has been more challenging compared to TILs. For instance, expression levels of immune checkpoints in blood-derived T cells is lower than in TILs (111). Additionally, circulating T cells expressing immune checkpoints could result from other pathogen-specific responses. To date, only two reports have used T-cell markers for enrichment of tumor-specific T cells from peripheral blood. In contrast to CD8^+^PD-1^−^ peripheral blood T cells, sorted CD8^+^PD-1^+^ cells from melanoma patients contained lymphocytes targeting neoantigens (28). Moreover, neoantigen specificities and TCR repertoires in CD8^+^PD-1^+^ cells from blood and melanoma tumors were very similar. More recently, isolation of circulating memory T cells based on CD62L and CD45RO expression enabled the identification of neoantigen-specific T cells (108). Enriching T cells based on marker expression is advantageous as no foreknowledge of T cell-specific reactivities or HLA restriction is required, thereby theoretically broadening its application to any patient. However, marker expression is variable among patients. Furthermore, the low frequency of marker-expressing cells demands an additional *in vitro* expansion step after sorting in order to achieve reasonable cell numbers for *in vitro* immunological screening assays, which could change the repertoire compared to the initial population. Although there is no direct evidence of this for marker-sorted cells in humans, mouse antigen-specific T cells among sorted CD8^+^PD-1^+^ TILs have been shown to decrease in frequency after *in vitro* expansion (132). Despite these challenges, this approach is attractive not only for neoantigen screening but also as a source of T cells for therapeutic applications such as ACT. Open questions regarding this strategy that still need to be addressed include: (i) which marker best recovers most of the neoantigen-specific T-cell repertoire, and (ii) whether the co-expression of multiple markers can improve enrichment based on single-markers.

Other enrichment strategies rely on the detection of the interaction between the TCR and its cognate pHLA complex. Staining of T cells with fluorescently-labeled pHLA multimers allows the simultaneous detection and sorting of pure antigen-specific populations, which can then be interrogated for validation of neoantigens in functional assays. Using this approach, multimer-enriched T cells from either PBMCs or fresh tumor digests have been used for validation of neoantigens derived from solid and hematological malignancies (99, 133, 134). Besides the disadvantages related to pHLA multimers mentioned in section Screening of Predicted or Eluted Minimal Neoepitopes, one that limits multimer-based enrichment of T cells is the fact that positive signals after multimer staining not necessarily determine functional T-cell responses (134–136).

Another frequently used enrichment strategy is *in vitro* sensitization (IVS), which exploits antigen-specific stimulation and expansion to increase the frequency of specific T-cell reactivities. The most frequently used approach of IVS involves the co-culture of either PBMCs or TILs, with or without irradiated feeders and a pool of candidate peptides in the presence of cytokine cocktails [usually combinations of interleukin (IL)-2, IL-7, IL-15, and IL-21]. Co-cultures are usually incubated for 10–14 days, after which the resulting T-cell populations can be screened for neoantigen recognition or for subsequent rounds of stimulations. A modified version of this approach involves the stimulation of TILs or PBMCs with autologous APCs electroporated or pulsed with TMGs or peptides (long or minimal epitopes), respectively, under similar culture conditions as the ones mentioned above. Alternatively, if the patient's autologous tumor cell line is available, it can be used instead of APCs for stimulation. These three strategies have proven to be useful for enrichment of neoantigen-specific T cells and for the subsequent validation of candidate neoantigens (86, 108, 137, 138). However, the simultaneous presentation of multiple epitopes during IVS may favor the enrichment of T-cell populations specific for immunodominant peptides, leading to underrepresentation of the true neoantigen-specific T-cell repertoire present in the starting population. To overcome the potentially biased enrichment of T cells and in order to detect the broader repertoire of neoantigen-specific T cells, a more reliable but also more cumbersome approach involves the stimulation of T cells with APCs pulsed with every single predicted minimal epitope for separate (91). It is important to note, however, that this strategy has been limited to tumors with low mutational load, or those whose neoantigen candidate list has been prioritized using *in silico* prediction algorithms.

The methods described in this section have been commonly used as single enrichment strategies. However, the combination of such enrichment strategies (e.g., marker-based selection and IVS) can result in highly enriched populations of neoantigen-specific T cells (108). Furthermore, recent efforts aim at combining enrichment methods, such as IVS with or without CD137-based T-cell selection, with other sensitive technologies such as TCRβ deep sequencing by NGS to screen for neoantigens (91, 139–141).

#### T-Cell Clones and TCR-Transduced Lymphocytes

The antitumor responses observed upon adoptive transfer of TILs targeting neoantigens has provided rationale to develop personalized T-cell therapies. However, the differentiated status of the administered cells has been associated with limited antitumor activity in mouse models, suggesting that TCR-gene engineered T cells could be more efficacious. This, combined with recent progress in the non-viral delivery of TCRs into PBLs (142), has made personalized neoantigen-specific TCR-gene engineering a true possibility.

The rapid identification of neoantigen-specific TCRs, a pre-requisite for the development of such therapies, can be performed through multiple strategies. First, T-cell clones can be established either from TILs, peripheral blood subsets or enriched populations (as described above) and can be screened for recognition of neoantigens. TCRα and TCRβ sequencing can be carried out from the neoantigen-specific clones to isolate the variable regions of the TCR. These can then be cloned into a vector of choice to transduce or transfect PBLs to express and test the specificity of the TCRs identified. This approach led to the rapid identification of six neoantigen-specific TCRs from two patients, including a high affinity HLA-II-restricted KRAS_p.G12D_-specific TCR in a recent report (30). However, the limited proliferative capacity of some clonotypes may result in a biased representation of the starting TCR repertoire.

A second approach to isolate neoantigen-specific TCRs uses the oligoclonality of specific tumor-resident TCR clonotypes as a surrogate to select for candidate neoantigen-specific TCRs that may have undergone clonal antigen-specific expansion. As exemplified in the work by Pasetto et al. the most frequent TCRβ clonotypes identified by TCRβ deep sequencing were selected as candidate tumor or neoantigen-specific lymphocytes and were paired with the most dominant TCRα sequences, leading to the cloning, expression and immunological testing of a few TCRα-β pairs (112). However, the inefficient allelic exclusion of TCRα chains during somatic recombination in T cells frequently leads to T cells harboring two TCRα sequences and this can hinder construction of the correct pairs. Alternatively, the TCRα sequence that pairs with the oligoclonal TCRβ clonotype selected can be identified using pairSEQ, a high-throughput strategy combining TCRα and TCRβ sequencing with statistical analysis to infer TCRα-β pairs from bulk PBLs or TILs (143). In addition, single-cell TCRα and TCRβ sequencing of cells directly isolated from the tumor can be carried out either using conventional sanger sequencing (112) or NGS (116), to identify TCRα-β pairs from TILs. Once the sequence of the selected TCRα-β pairs are identified, T cells can be screened for recognition of candidate epitopes in functional assays that require autologous target cells (116). Using this approach, Pasetto et al. generated PBLs expressing 68 TCRα-β pairs derived from melanoma-resident CD8^+^PD-1^+^ T cells from 10 patients and successfully identified 9 neoantigen-specific TCRs. Furthermore, recently, single-cell transcriptomics has enabled to couple specific TCRα-β sequences to specific differentiation and functional traits. Although this technology has not yet been exploited to isolate neoantigen-specific TCRs from TILs, it could further improve our understanding of the functional and phenotypic traits of TILs and the accuracy of existing biomarkers to select for candidate neoantigen-specific lymphocytes. The major limitation of this approach is the high amount of TCR clonotypes that can be retrieved from all the sequencing data. Hence, high-throughput platforms to gene-engineer and test the specificity of such high number of TCRs is currently a matter of extensive research (144).

## Concluding Remarks

Virtually all cancers harbor genetic alterations, some of which can give rise to mutated, non-self peptides presented by HLA molecules and elicit T-cell responses, referred to as neoantigens. Recent data suggests that neoantigen-specific lymphocytes can be detected in the vast majority of cancer patients, regardless of their tumor mutation burden. Moreover, they appear to have a central role in the clinical activity of cancer immunotherapies. Thus, neoantigens have emerged as promising targets for personalized immunotherapies. However, mounting evidence suggests that only a small fraction of the NSM identified by tumor WES are actually immunogenic. While inherent difficulties can limit neoantigen identification, such as tumor heterogeneity or as a result of *holes* in the TCR repertoire, the success or failure of neoantigen identification is, in great part, determined by the identification of candidate neoantigens and the immunological screening assays required to identify *bona fide* neoantigens, all with their own advantages and disadvantages.

Whilst *in silico* peptide prediction strategies have led to the identification of neoantigens, they can inaccurately predict peptides, and they are not efficiently trained to identify HLA-II neoantigens. Immunopeptidomics can be used to discover novel neoantigens or validate those obtained using *in silico* peptide predictors, but MS based identification of peptides is limited by its current sensitivity and by the fact that some peptides may never be detected using this approach. To date, the safest, albeit, the most laborious, and costly strategy to identify neoantigens requires the unbiased screening of all neoantigens identified using TMGs or PPs, as demonstrated by the growing number of neoantigens identified using this approach in the last five years. This strategy has provided a broader idea of the frequency of neoantigen reactivities in cancer patients and is capable of detecting CD4^+^ T-cell responses targeting neoantigens, which may be important to develop effective treatments. Moreover, the specific immunological screening method and read-outs selected, as well as the choice of effector population screened can also greatly impact on neoantigen identification.

Thus far, clinical trials testing vaccines targeting neoantigens have demonstrated they are safe and well tolerated, and personalized T-cell based therapies targeting neoantigens have shown antitumor responses in selected cases. However, whether individualized immunotherapies targeting neoantigens can mediate effective antitumor responses in a broader patient population, remains an open question. Despite all the technological innovation and development of novel screening assays, the rapid and precise identification of the *bona fide* neoantigens in any given patient remains a major hurdle that will need to be overcome to translate the potential of neoantigen targeting into effective therapies for patients with cancer.

## Author Contributions

AG conceived the review. All authors listed made a substantial, direct, and intellectual contribution to the work. Figures were created by AG-G with BioRender.com.

### Conflict of Interest Statement

AG reports receiving funding from Novartis, Roche and EMD Serono, has received speaker honoraria from Roche, and has consulted for Achilles Therapeutics, Neon Therapeutics and PACT Pharma. AG is co-inventor in a patent application regarding the isolation of T cells and T-cell receptors targeting neoantigens from tumor and peripheral blood (US Application number 15/567,157). The remaining authors declare that the research was conducted in the absence of any commercial or financial relationships that could be construed as a potential conflict of interest.
